# Neuroprotective Effect of Nosustrophine in a 3xTg Mouse Model of Alzheimer’s Disease

**DOI:** 10.3390/ph16091306

**Published:** 2023-09-15

**Authors:** Iván Carrera, Lola Corzo, Olaia Martínez-Iglesias, Vinogran Naidoo, Ramón Cacabelos

**Affiliations:** EuroEspes Biomedical Research Center, Institute of Medical Science and Genomic Medicine, 15165 Bergondo, Corunna, Spain; analisis@euroespes.com (L.C.); epigenetica@euroespes.com (O.M.-I.); neurociencias@euroespes.com (V.N.); rcacabelos@euroespes.com (R.C.)

**Keywords:** Alzheimer’s disease, 3xTg-AD, nosustrophine, porcine brain extract, neuroprotection

## Abstract

Neurodegeneration, characterized by the progressive deterioration of neurons and glial cells, is a feature of Alzheimer’s disease (AD). The present study aims to demonstrate that the onset and early progression of neurodegenerative processes in transgenic mice models of AD can be delayed by a cocktail of neurotrophic factors and derived peptides named Nosustrophine, a nootropic supplement made by a peptide complex extracted from the young porcine brain, ensuring neuroprotection and improving neuro-functional recovery. Experimental 3xTg-APP/Bin1/COPS5 transgenic mice models of AD were treated with Nosustrophine at two different early ages, and their neuropathological hallmark and behavior response were analyzed. Results showed that Nosustrophine increased the activity of the immune system and reduced pathological changes in the hippocampus and cortex by halting the development of amyloid plaques, mainly seen in mice of 3–4 months of age, indicating that its effect is more preventive than therapeutic. Taken together, the results indicate the potent neuroprotective activity of Nosustrophine and its stimulating effects on neuronal plasticity. This study shows for the first time an effective therapy using nootropic supplements against degenerative diseases, although further investigation is needed to understand their molecular pathways.

## 1. Introduction

Neurodegenerative diseases pose a major therapeutic challenge due to the complex mechanisms of neuronal cell death. Memory and motor skills are gradually lost as a result of the most severe neurodegenerative disorders, such as Alzheimer’s disease (AD), Parkinson’s disease (PD), and Amyotrophic lateral sclerosis (ALS). The last ten years have seen a clear increase in scientific evidence from experimental studies that certain neurotrophic factors and growth peptide factors, derived from various neuroprotective protein sources, can restore neuronal function, improve behavioral deficits, and extend the survival rate of neuronal cells in animal models [1] and patients [2]. Nootropics [3], which are dietary supplements that are eaten primarily to increase cognitive function, are another name for these cognitive enhancers. Due to their strong neurobioactive effects [4], natriuretic peptides, in particular the urodilatin (CDD/ANP-95-126), atrial (ANP), brain (BNP), C-type (CNP), and Dendroaspis natriuretic peptides (DNP), are starting to be researched in depth. The majority of these hormonal/peptide variables serve as effective neuropeptides in the brain even though they govern particular metabolic bodily activities. Additionally, these neurotrophic peptides have been isolated from porcine brain along with other well-known factors, such as the endothelial cell growth factor (ECGF), fibroblast growth factor (FGF), nerve growth factor (NGF), brain-derived neurotrophic factor (BDNF), neurotrophin-3;-4/5 (NT-3;NT-4/5), ciliary neurotrophic factor (CNTF), insulin-like growth factor (IGF), transforming growth factor (TGF-), and cholinergic neuronal differentiation factor (CNDF), have also been isolated from porcine brains on the basis of their potent neuroactive effects [5]. These neuropeptides work on the central nervous system to preserve neurons and enhance learning and memory in mice with scopolamine-induced impairment. They were extracted from the pig brain as a cerebroprotein hydrolysate extract [1]. In vitro [6] and in vivo [7] reports of the neuroprotective benefits of pig brain extract have also been made. As a result, porcine brain extract appears to be a strong contender for a brand-new neurotherapeutic source.

The neuro-therapeutic approach needs to shift to prioritize neuroprotection, reduce neuroinflammation, and stop cell death in light of the current understanding of the neuronal degenerative process. Although there are currently no effective medications to prevent the onset and progression of neurodegenerative illnesses, neuroprotective factors, which combine neuropeptides from the brain with neurotrophins, may be prospective therapeutic targets [8]. Neuropathological diseases’ degeneration is slowed down by brain-derived trophic peptides utilized to address endogenous neurotrophic factor deficiencies [9]. These peptides promote cell survival and potential cell growth in vitro and in vivo, as well as other positive actions that are related to protecting neurons against oxidative stress-induced degeneration [10]. Additionally, neurotrophic substances may enhance neuronal metabolism and boost cellular performance, which could restore synaptic plasticity through the creation of new axons, improve cognitive function by bolstering neuroconnec-tivity, and improve long-term memory [11]. There is currently no cure for slowing the disease’s progressive deterioration of the affected brain regions, and conventional treatment usually focuses on palliative medication to delay the onset of dementia. However, many patients do not respond to this kind of pharmacological therapy approach, which has grave health risks from a pharmacogenomics standpoint [12]. Along with the list of medications authorized by the Food and Drug Administration (donepezil, memantine, tacrine, rivastigmine, galantamine), most candidate strategies fall into six major categories: (i) inhibitors of cholinesterase and neurotransmitter regulators, (ii) anti-amyloid beta (Aβ) treatments (Aβ active and passive immunotherapy with vaccines), (iii) anti-tau treatments, (iv) pleiotropic products of natural origin, (v) novel epigenetic intervention, and (vi) combination of more than one medication or therapy [13]. As a result, pharmacogenomic approaches will directly contribute to improved medication treatment outcomes in individuals with AD or other similar illnesses [14]. One of these innovative pleiotropic bioproducts is Nosustrophine, a nootropic supplement made from specific parts of young pig brains using non-denaturing biotechnological methods [15] and administered in the diet as shown in Table 1. The goal of this sophisticated formulation is to boost endogenous neuropeptide synthesis and release by activating neuro-enzymatic processes. In the current study, we discovered that soluble neuropeptides and neurotrophins from pig brain retard the progressive development of AD pathological hallmarks in the 3xTg-APP/Bin1/COPS5 (3xTg-AD) mouse model of AD based on the interaction between natriuretic peptides and AD-associated pathways [16,17]. Experimental mice models of 3 and 9 months of age were selected based on the early stages of development as they represent the stages during which AD has started and is progressing, but without severe neuropathology. The importance of the present study is major in the field of neurodegenerative diseases since Nosustrophine contains BDNF and corticotropin-releasing factors, among other bioactive agents [15] that reduces the cytotoxic effects of neurodegeneration while promoting an improvement in cognitive function, we aimed to demonstrate that neuropeptides derived from the porcine brain may be protective against neurodegenerative diseases such as AD.

## 2. Results

### 2.1. Biochemical Effect of Nosustrophine in Serum from 3xTg-AD Mice

**Figure 1:**
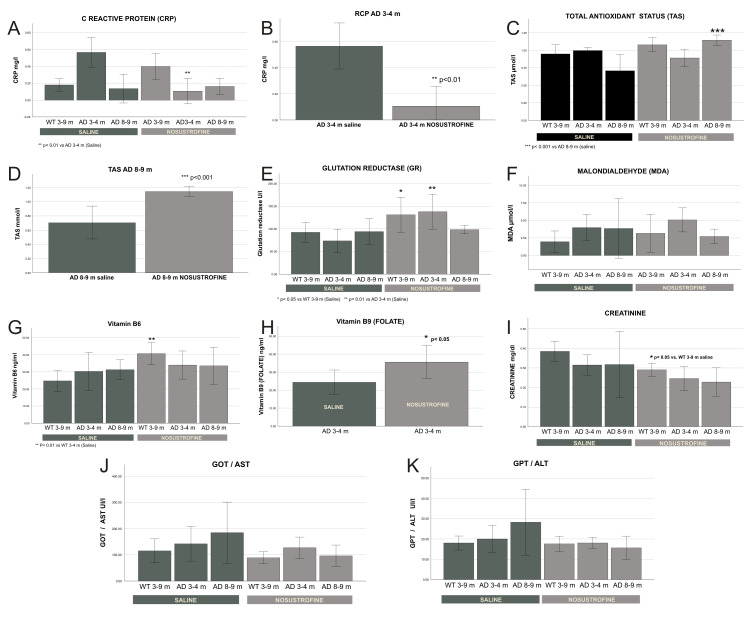
Biochemical regulation of Nosustrophine in 3xTg-AD mice serum. Wild-type (WT) and AD-mice were treated with Nosustrophine. Most of the parameters analyzed show a significant correlation between an improved biochemical neuroprotection and mice treated with Nosustrophine, mainly in the early stages. (**A**,**B**): Inflammation response: Nosustrophine decreased C reactive protein (CRP) in young AD-mice. (**C**–**E**): Antioxidant status improves with Nosustrophine: TAS increased in adult AD-mice (non-significant for WT) and GR increased mainly in WT and young AD-mice. (**F**): In lipid peroxidation (MDA), as marker of oxidative stress, no significant results were found. (**G**,**H**): Vitamin B6 levels increased in WT mice and Folate levels were higher in young AD-mice, both treated with Nosustrophine. (**I**–**K**): Nosustrophine tends to decrease creatinine concentration and transaminases activity (GOT and GPT) in all groups being only significant for creatinine in WT mice. Significant values are represented as indicated * (*p* < 0.05), ** (*p* < 0.01) and *** (*p* < 0.001); Error bars: 95% CI. Data are expressed as mean ± standard error of the mean (SEM).

To evaluate the biochemical effects of Nosustrophine in 3xTg-AD mice, we chose different markers associated with AD etiopathology. We analyzed the levels of reactive C protein (RCP) as a biomarker of inflammation. To assess the oxidative stress, we measured total antioxidant capacity (TAS), glutathione reductase (GR) activity, an antioxidant enzyme, and malondialdehyde (MDA) levels, an indirect measurement of lipid peroxidation and the most studied marker of oxidative damage. B-complex vitamins, specifically vitamin B6 and B9 (folate), were selected due to its proven association with cognitive performance [15]. Other AD-related biomarkers such as minerals (phosphorous, calcium, magnesium, and iron) were also assessed. The administration of Nosustrophine caused a significant reduction in the RCP concentration at two different early ages 3xTg-AD mice (Figure 1A,B) compared to other treatment groups. With respect to Nosustrophine-derived antioxidant effects, TAS increased in older 3xTg-AD mice (Figure 1C,D) and GR activity increased in younger 3xTg-AD mice (Figure 1E), but there were no significant changes in post-treatment lipid peroxidation (MDA) levels (Figure 1F). We also found a significant increase in the levels of vitamin B6 in wild-type mice (Figure 1G) and vitamin B9 (folate) in young 3xTg-AD mice (Figure 1H). No significant effects were observed in terms of the minerals analyzed, although there was a tendency for higher calcium and magnesium levels and lower serum iron levels in 3xTg-AD mice than in saline-treated mice. The decrease in creatinine concentration (Figure 1I) and liver enzyme activity (GOT and GPT transaminases; Figure 1J,K) after Nosustrophine administration, both in wild-type mice and in 3xTg-AD mice, indicated the absence of Nosustrophine toxicity in the kidney and liver.

### 2.2. In Vivo Experimentation

**Figure 2:**
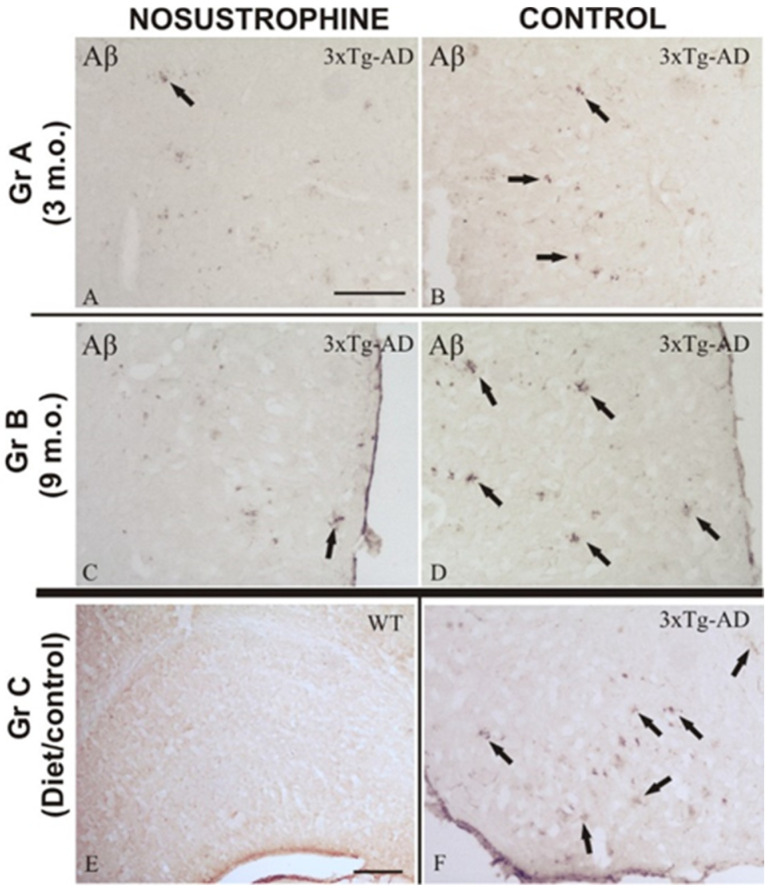
Progressive regulation of Aβ deposition in 3xTg-AD mouse brains treated with Nosustrophine. Comparative photomicrographs showing representative cortical and hippocampal brain sections of 3–9-month-old EB/3xTg-AD mice immunostained for Aβ. Images are presented according to the age group. (**A**–**D**) Images of mice given Nosustrophine show early A-immunoreactive plaques in the cortical and hippocampus regions, whereas control sections display multiple A-immunoreactive plaques with a compact morphology at various stages; (**E**) nosustrophine-treated wild-type mice’s control brain slice, demonstrating no negative effects; (**F**) cortical slices of the control brain of EB/3Tg-AD transgenic mice are heavily marked by A-immunoreactive plaques that have a large diameter and a compact central core. Scale bar: 100 μm.

**Figure 3:**
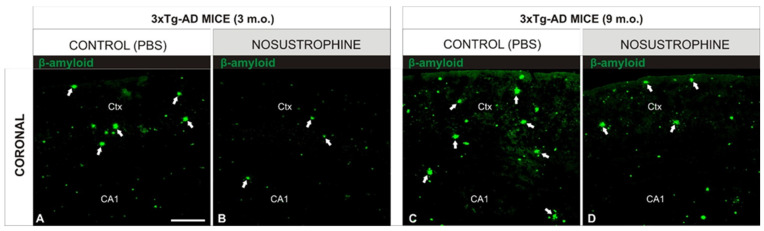
Nosustrophine modulates cortical density of β-amyloid plaques in early stages of AD mouse models. Comparative photomicrographs showing representative 3- and 9-month-old EB/3xTg-AD mouse brain sections stained by immunofluorescence antibody against β-amyloid. (**A**,**C**) Images showing the progressive accumulation of β-amyloid immunoreactive plaques in cortical regions of EB/3xTg-AD transgenic mice with no treatment (PBS); (**B**,**D**) in contrast, EB/3xTg-AD transgenic mice treated with Nosustrophine show a lower density of β-amyloid immunoreactive plaques in the early and middle stages of brain development. For abbreviations, see list. Scale bar: 100 μm.

**Figure 4:**
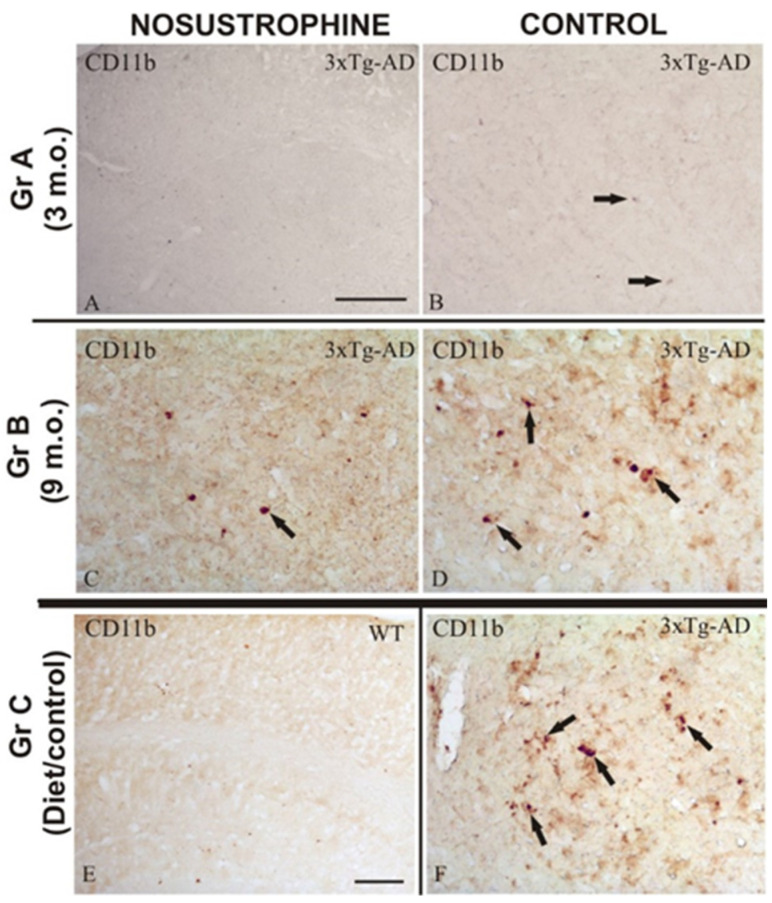
Modulation of reactive immune system in AD mouse brains treated with Nosustrophine. Comparative photomicrographs showing representative brain regions of 3–9-month-old 3xTg-AD mouse brain sections immunostained for the lymphocyte marker CD11b. Images are presented according to the age group. (**A**–**D**) Images are shown based on the age group. Images of the interior neocortical and hippocampus layers of 3xTg-AD transgenic mice depicting the increasing accumulation of CD11b-immunoreactive cells as an inflammatory response. In contrast, Nosustrophine-treated 3xTg-AD transgenic mice exhibit a low density of CD11b-immunoreactive cells at early and late phases of brain development; (**E**) nosustrophine-treated wild-type mice’s control brain slice, demonstrating no negative effects; (**F**) anatomical image of the control brain of 3xTg-AD transgenic mice demonstrating the abundance of inflammatory CD11b-immunoreactive cells in the damaged brain regions. Scale bar: 100 μm.

**Figure 5:**
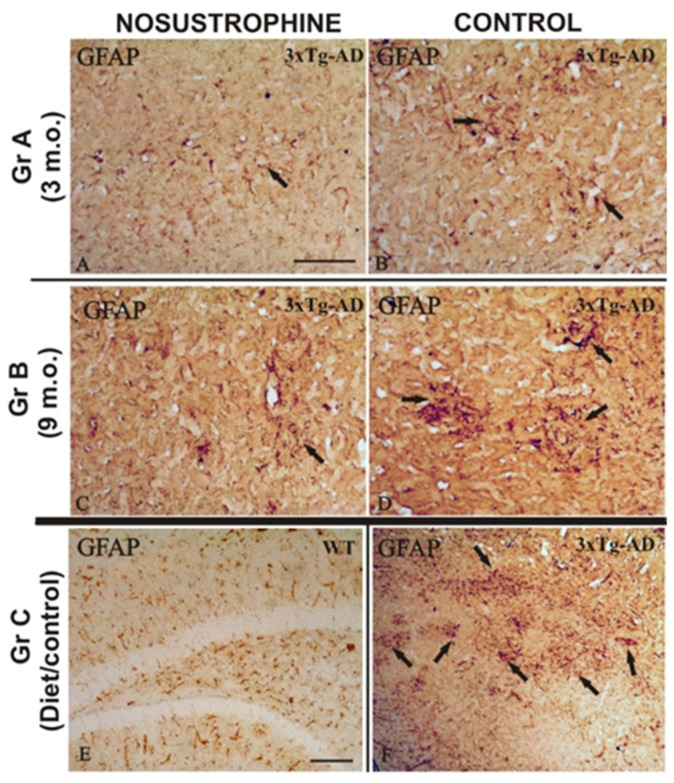
Nosustrophine regulate astrogliosis in AD mouse brains. Comparative photomicrographs showing representative brain regions of 4–9-month-old EB/3xTg-AD mice brain sections immunostained for GFAP. Images are presented according to the age group. (**A**–**D**) Images showing the progressive accumulation of dystrophic reactive astrocytes from the dentate gyrus to the external layers of the neocortex. Note the contrast in astrocytosis density between Nosustrophine-treated mice and controls, where numerous immunoreactive GFAP clusters are observed; (**E**) control brain section of wild-type mice treated with Nosustrophine, showing no adverse effects; (**F**) control brain section of EB/3xTg-AD transgenic mice showing numerous inflammatory GFAP-immunoreactive cell clusters in the cortical and hippocampal brain areas. Scale bar: 100 μm.

**Figure 6:**
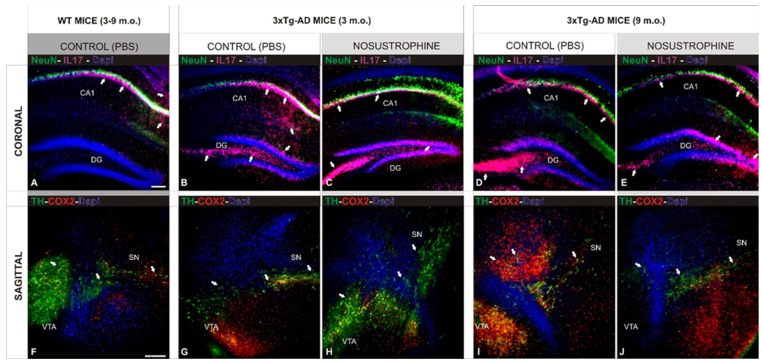
Nosustrophine effect on neuropathological hallmarks in AD mouse models. Comparative photomicrographs showing representative 3 and 9-month-old EB/3xTg-AD mouse brain sections, early and middle stages of brain development, stained by antibodies against NeuN (neuronal nuclei), Cox2 (apoptosis), IL17 (proinflammatory cytokines), and TH (catecholaminergic neurons). (**A**–**E**) Images of hippocampal regions showing the progressive accumulation of AD pathological markers in control (not treated) mice; (**B**,**D**) when compared with wild-type mice (**A**) and 3xTg-Ad treated with Nosustrophine (**C**,**E**); (**F**–**J**) Images of midbrain regions showing the massive accumulation of AD pathological markers in control (not treated) mice; (**G**,**I**) when compared with wild-type mice (**F**) and 3xTg-Ad treated with Nosustrophine (**H**,**J**). Scale bar: 100 μm.

To evaluate amyloid plaque burden, we used immunohistochemistry with specific antibodies to evaluate amyloid burden (β-amyloid), astrogliosis (GFAP), immune activation (IL-17, CD11b), apoptosis induction (Cox-2), neuronal differentiation (TH), and development (NeuN) (Table 2) in all mice (Figure 2, Figure 3, Figure 4, Figure 5 and Figure 6). AD markers showed classic hallmarks of neurodegeneration in all transgenic mice, including Nosustrophine-treated mice and control AD mice at 8–9 months of age (Figure 2, Figure 3, Figure 4, Figure 5 and Figure 6) although at different densities. Brain immunocytochemical analysis of 3- and 9-month-old EB/3xTg-AD transgenic mice revealed damaged neurons mostly in the neocortex, dentate gyrus, and in the sub-granular zone of the hippocampus. No pathological hallmarks were observed in the corresponding areas in saline treated wild-type mice.

### 2.3. The Neurotrophic Action of Nosustrophine Induces Neuronal Development

**Figure 7:**
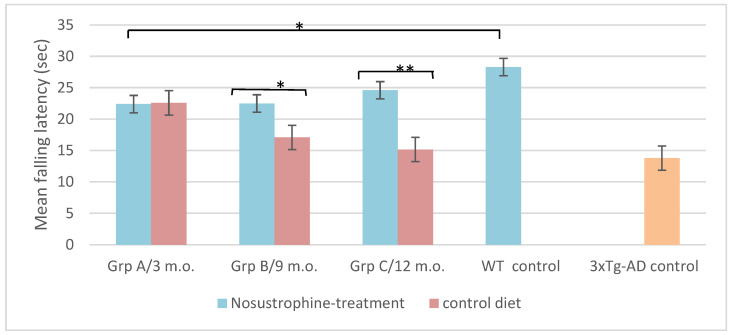
Nosustrophine improves motor coordination in AD mouse models. Rotarod results of motor coordination and balance test of EB/3xTg-AD transgenic mice during four weeks. Notice the progressive decrease in rotarod performance that is directly related to the age of the mice. Latency to fall on the rotarod apparatus was significantly improved in mice treated with Nosustrophine, mainly in later stages as groups B (* *p* < 0.05) and C (** *p* < 0.01). Significant values represent means of latency of all mice in each group/trial and are expressed as standard error of the mean (±SEM).

Nosustrophine-treated 3xTg-AD mice showed significant neuroprotective effects, preserved the morphologic organization of the neurogenesis areas (lateral ventricles and the dentate gyrus of the hippocampus) and a lower density of β-amyloid plaques, respectively (Figure 2A,C). It is important to emphasize the maintenance of the cytostructure of neurons and tracts of the affected areas, as well as the optimal neuronal density in the ventricular ependyma, structures that were strongly affected during the neuropathological development of the disease. Although the NeuN specific biomarker used to address the effect of Nosustrophine on neuroprotection in the dentate gyrus of mice showed a notable effect on treated mice brains (Figure 6), the cytoarchitecture of the dentate gyrus also showed a compact histological conformation of cell layers (Figure 2) of the Nosustrophine treatment. However, in control 3xTg-AD mice (3–9 m.o.), these cell layers thickness were reduced and the altered cytoarchitecture of the neurogenesis areas, indicating a lower neurotrophic level (Figure 2B,D and Figure 6B,D,G,I). As a control in wild-type mice, Nosustrophine showed no adverse effects either at the histological (Figure 2, Figure 3, Figure 4, Figure 5 and Figure 6) or behavioral levels (Figure 7).

### 2.4. Nosustrophine Disrupts the Progressive Neuropathology in 3xTg-AD Mice

An important pathological feature of AD is irreversible neuronal degeneration that triggers the activation of apoptotic markers and subsequent necrosis, resulting in massive neuronal death. In this study, Nosustrophine reduced neurodegeneration by maintaining neuronal density in the regions affected by β-amyloid plaques (Figure 2 and Figure 3). The anti-degenerative effect of Nosustrophine was observed in the cortex and CA1 region of the hippocampus (Figure 2 and Figure 3). There were approximately 10–15% more β-amyloid plaques in 3-month-old mice (Figure 2B and Figure 3A) and 20–30% in 9-month-old mice (Figure 2D and Figure 3C), than in saline-treated wild-type controls (Figure 2E). Compared to the Nosustrophine-treated groups, control 3xTg-AD mice showed extensive Aβ deposition in the neocortex and hippocampus that increased over time (Figure 2F). In contrast, the Nosustrophine treated 3xTg-AD mice showed reduced amyloid deposition that was continuously sustained during development and that were significantly fewer in density during the early (3 m.o.) and mid (8–9 m.o.) stages (Figures 2A,C and 3B,D) compared to 3xTg-Ad controls. Analysis of 3-month-old mice showed that untreated transgenic mice displayed different levels of β-amyloid immunoreactivity in the molecular layer of the dentate gyrus (Figure 2B) and in the external cortical layers (Figure 3A), whereas Nosustrophine-treated mice had slightly lower (<62%) density of β-amyloid plaques (Figure 2A and Figure 3B). As they matured, 3xTg-AD mice treated with Nosustrophine showed a significantly reduced density of β-amyloid plaques compared to control groups at 9 months old (Figure 2D and Figure 3C).

### 2.5. Nosustrophine Reduces Inflammatory Activity in Cortical and Hippocampal Regions

Bin1/Cops5/App transgenic mice exhibit a highly reactive neuroinflammatory process in the cortex and hippocampus due to β-amyloid plaque-induced neuropathological lesions. Therefore, in this study we analyzed whether Nosustrophine improves neuronal survival by inhibiting the activation of astrogliosis and reactive microglia in the neocortex and hippocampus. Immunohistochemistry using specific antibodies against microglia (CD11b) and astroglia (GFAP), in all mice groups (Figure 4 and Figure 5), showed that Nosustrophine significantly reduced the density of activated microglia (Figure 4A,C) and astrogliosis (Figure 5A,C) in 3xTg-AD mice. In contrast, results show that 3–9 m.o. AD mice presented numerous pathological hallmarks associated with reactive inflammation (Figure 4B,D) and derived response as astrogliosis (Figure 5B,D) throughout the main affected brain regions. In particular, there was a lower density of immunoreactive CD11b cells in the brain of Nosustrophine-treated mice, especially at the hippocampal regions affected by fibrillar amyloid-containing plaques (Figure 4A,C). On the other hand, the distribution of GFAP immunoreactive glial cells in both early ages AD mice treated with Nosustrophine showed a few scattered GFAP-reactive clusters, mainly in the hippocampal layers (Figure 5A,C). This contrasted with numerous dystrophic reactive astrocytes observed in different hippocampal areas in control mice (Figure 5B,D). Similarly, the pattern of neuroinflammation was observed in the retrosplenial cortex, where a few GFAP-reactive clusters were observed in Nosustrophine-treated mice (Figure 5A,C) compared to their profuse presence in the other experimental groups (Figure 5B,D). This immunoreactive labeling pattern of CD11b (Figure 4E) and GFAP (Figure 5E) positive cells were scarce in the wild-type mouse brain.

### 2.6. Neuroprotective Effect of Nosustrophine on 3xTg-AD Mice Neuropathology

To address the Nosustrophine effect on neuropathological hallmarks in 3xTg-AD mouse models, in the early and middle stages of brain development (3 and 9-month-old), multiple immunofluorescence markers were tested in both coronal (hippocampus) and sagittal (midbrain) sections of the brain. Control saline-treated 3xTg-AD mice had a low density of NeuN-positive neurons at both 3- (Figure 6B) and 9- (Figure 6D) months-old, compared to wild-type and 3xTg Nosustrophine-treated mice (Figure 6C,E). The high density of IL17-positive cells in control groups also indicated extensive inflammation. (Figure 6B,D), contrasting with their low density in the Nosustrophine-treated groups of both ages (Figure 6C,E). The decrease in the density of catecholaminergic neurons in the midbrain is one of the main pathological hallmarks of AD, and was observed in saline-treated 3xTg-AD mice (Figure 6G,I). In the same brain region in mice treated with Nosustrophine, TH-positive cells (a marker of dopaminergic neurons affected in the degeneration process) were at a higher density (Figure 6H,J), although showing differences between both developmental stages. Early stage mice (3 m.o.) treated with Nosustrophine showed a massive population of catecholaminergic neurons at the midbrain (Figure 6H), although this treatment effect was moderate in the mid-developmental stage (Figure 6J). The degenerative pattern of these midbrain neurons was identified by Cox2-immunoreactivity, an indirect apoptotic marker (Cox2), proinflammatory cytokine (IL17) and a marker of dopaminergic neurons (TH). A notable difference in the density of Cox2-labeled cells was observed between untreated (saline) and treated (Nosustrophine) mice brain at 3 months old (Figure 6G,H), while little to no difference in Cox2 cell density was observed at middle stages (Figure 6I,J). These coronal (Figure 6A) and sagittal (Figure 6F) brain sections from wild-type mice showed similar patterns to the early stages of AD in our mice model of AD (Figure 6H).

### 2.7. Nosustrophine Modulate Motor Coordination Performances in 3xTg-AD Mice

The rotarod test was performed using mice from all groups to evaluate the locomotion integrity and motor coordination during the entire developmental process (Figure 7). Motor coordination tests have shown an inverse correlation between 3xTg-AD mice age and the time spent on the accelerating rotarod. Latency to fall on the rotarod apparatus was significantly aggravated in 3xTg-AD mice of control groups (*p* < 0.05), while both Nosustrophine-treated mice groups display similar results to wild-type mice with no significant difference between trials (Figure 7). These data suggested that the increase in exposure time to the extract resulted in an effective improvement in brain functions that are linked to motor coordination and balance, which had significantly decreased in 3Tg-AD mice.

## 3. Discussion

The primary pathogenic consequence of AD in the brain is irreversible neuronal degeneration, which sets off necrotic and apoptotic processes and results in widespread neuronal death [18]. The current experimental investigation shows that Nosustrophine ex-tract prevented the growth of -amyloid plaques, astrogliosis, and neuroinflammation in afflicted brain areas, thereby slowing the degeneration of neurons in 3Tg-AD mice. The anti-degenerative effect of Nosustrophine was not-able in the affected regions (cortex and hippocampus), where -amyloid plaque density was significantly reduced, similar to the brain regions affected by astrogliosis and neuroinflammation. The results of the distribution of -amyloid plaques and the adjacent neuronal structure in all groups of mice treated. Numerous lines of research revealed that the pathophysiology of AD is significantly influenced by the imbalance of free radicals [19], neurotrophic factors [20], and activated inflammatory cytokines [21]. Active antioxidants, however, have the ability to lessen this impairment by boosting the activity of scavenger enzymes [22]. Prior research has shown that upregulating scavenger enzymes can inhibit both diffuse and localized neuroinflammation processes [23]. By increasing neuroprotective activity in the cortex and hippocampus of 3xTg-AD mice, lowering the number of -amyloid plaques, and reducing IL-17 levels, our results demonstrated that Nosustrophine could slow the progression of AD effects. This effect may have been caused by the direct action of neurotrophic factors found in the porcine brain extract. Through the analysis of multiple specific metabolic indicators including RCP, TAS, GR, and MDA, among others, biochemical data drawn from serum of mice in all treatment groups validated this hypothesis. RCP is a plasma protein that circulates and rises in concentration in response to inflammation (acute phase protein). It is mostly produced in the liver in response to IL-1 [24], and its availability enables the assessment of the degree and severity of inflammation, as well as the follow-up and prognosis of patients with neuroinflammatory disorders [25]. Young 3Tg-AD mice treated with Nosustrophine had significantly lower RCP concentrations, demonstrating the anti-inflammatory potential of this compound in these animals. As a result, Nosustrophine may act in the early stages of the disease (or in cases of mild cognitive impairment) by attenuating the inflammatory process, perhaps delaying the progression of the disease to more advanced stages. 

In the present study, the levels of antioxidants/oxidants were measured in mice serum. TAS and GR levels increased in 3xTg-AD mice treated with Nosustrophine, compared to the group treated with saline, reflecting the antioxidant and consequently, strong neuroprotective effect, of the extract in 3xTg-AD mice. This outcome was true for both GR and TAS in older 3Tg-AD mice as well as younger 3xTg-AD animals. However, although we did notice a minor increase in MDA levels in young 3xTg-AD (3–4 m.o.) mice, there were no appreciable alterations in lipid peroxidation as determined by MDA levels following therapy. In contrast, a trend to decline was seen in 8–9 month old 3xTg–AD mice, but this was not seen in the group treated with saline, indicating that Nosustrophine has an age-dependent impact. According to our most recent research, the extract protects 3xTg-AD mice from oxidative damage by enhancing antioxidation pathways while having no appreciable impact on peroxidation (a direct result of oxidative stress), indicating Nosustrophine’s neuroprotective properties. When the Nosustrophine extract was administered to young 3xTg-AD mice four weeks later, we discovered a considerable rise in folate (B9) levels. Additionally, there was a considerable rise in the serum levels of vitamin B6 in wild-type mice. These findings point to a potential Nosustrophine preventative effect on cognitive impairment in the early stages of the disease. Numerous elements of cognitive function have been linked to these B vitamins in cross-sectional and longitudinal investigations, raising the potential that even subclinical variations in nutritional status may have a modest impact on these aspects [26]. Additionally, preliminary data suggests that supplementation can help older persons function better cognitively. The development and maintenance of brain cells is accomplished by B vitamins. People with mild cognitive impairment who took a vitamin B supplement demonstrated a delay in the acceleration of brain atrophy toward AD compared to those who did not [27]; its impact was linked to a reduction in homocysteine levels [28]. Although there was a trend for raised calcium levels in both Nosustrophine-treated groups of 3xTg-AD mice and for magnesium levels in older 3xTg-AD mice, our findings did not show a significant change in the blood levels of calcium, magnesium, or iron after the administration of Nosustrophine (8–9 m.o.). Additionally, there was a propensity for lower iron levels, particularly in young wild-type and 3xTg-AD mice. The magnesium concentrations of triple-transgenic AD animals were lower than those of wild-type mice, supporting earlier studies [29]. Since many years ago, AD has been linked to altered met-al-mineral homeostasis. Mg, Cu, Zn, Fe, and Se plasma levels are considerably lower in AD patients than in controls [30]. The reduction in creatinine concentration following Nosustrophine treatment, in both wild-type and 3xTg-AD mice, supports the extract’s renal safety. Moreover, the results also show a decrease in the activity of liver transaminases enzymes (GOT and GPT) in wild-type and 3xTg-AD mice, confirming the safety of Nosustrophine at the hepatic level. These mineral elements were found in Nosustrophine extracts as reported previousy [15]. 

It is well known that neurotrophic drugs promote the maturation and growth of neurons. Different neuronal populations, according to the “neurotrophic hypothesis,” depend on these substances for normal survival, but their concentrations are insufficient to support all of the neurons produced during the early stages of development [31,32]. Since the refined pig brain extract has historically been thought of as a food supplement to enhance brain function, its many components, which contain all these growth-promoting and neurotrophic elements identified, may elicit therapeutic action against AD pathology [33,34]. Due to their potential neuroprotective effects, polypeptides derived from porcine brain in particular may increase the activities of several scavenger enzymes such as superoxide dismutase, catalase, and glutathione-related enzymes [33], making them a priority focus of research in the treatment of degenerative diseases. As a result, in addition to each polypeptide’s individual bioactivity, which may include regulating circadian rhythm (BDNF), promoting peripheral nerve regeneration (NGF), or stimulating growth hormone secretion (GH-RH), their combined action primarily supports the regulation of growth, maintenance, proliferation, and survival of developing and mature neurons by intracellular signaling through specific receptors [35]. 

The refined pig brain extract used in the present study was tested by veterinary controls in order to avoid any transmissible diseases such as the bovine spongiform encephalopathies (BSE). Although the presence of infectivity in lymphoid tissues, striated muscles, and peripheral nerves in bovine PrP transgenic mice demonstrate the ability of BSE-derived agents to replicate efficiently in various peripheral tissues in pigs, no prion transmission has been reported in pigs following oral BSE exposition [36]. Therefore, the prevalence a transmissible disease throughout the pig brain extract is extremely low and prudently blocked by continuous veterinary control analysis. 

Continuous administration of Nosustrophine reduces and mitigates the neuropathological hallmarks in the affected brain regions of EB/3xTg-AD mice, when compared to controls. We compared the development of Aβ pathology at different developmental stages between treated and untreated EB/3xTg-AD transgenic mice, together with other neuro-immunological biomarkers such as GFAP and CD11b. These immune biomarkers, as well as the extracellular Aβ deposits in the cortex, are already apparent at four months of age. Therefore, in our study the neuroprotective effect of Nosustrophine treatment showed to be crucial on the maintenance of neurogenesis observed in the dentate gyrus of EB/3xTg-AD transgenic mice, suggesting a pivotal role of the brain derived peptides in the induction and regulation of neurogenesis through different mechanisms. Neurotrophic actions of brain-derived peptides are essential for the differentiation and early maturation of neuronal cell layers [37,38], triggers osteogenic differentiation of rat bone marrow-derived mesenchymal stem cells [39,40], demonstrating its efficiency as a potential biosource of tissue regeneration. Therefore, similar beneficial effects were observed when Nosustrophine was supplemented in the diet of AD mouse models, demonstrating not only the cytoarchitecture maintenance of neurogenesis layers within the periventricular brain areas but also the reduction in the pathologic degenerative hallmarks of the disease.

## 4. Materials and Methods

### 4.1. Mouse Models

3xTg-AD (*App*/*Bin1*/*Cops5*) mice were generated and used in the present research study. This triple transgenic mouse AD model closely resembles the pathophysiology of the human brain by overexpressing the Swedish mutation of APP (human amyloid precursor protein), BIN1 (bridging integrator 1, AMPH2), and COPS5 (COP9 constitutive photomorphogenic homolog subunit 5, Jab1). Prior to breeding the transgenic colony, DNA constructions and transgenic production processes were sequence-verified and previously published [15]. All experimental mice procedures were conformed to the guidelines established by the European Communities Council Directive (86/609/EEC), the EU Directive 2010/63/EU, and the Spanish Royal Decree 1201/2005 for animal experimentation and were approved by the Ethical Committee of the EuroEspes Biotechnology Research Centre (Permit number: EE/2017-127). 

### 4.2. Biochemical Characterization of Nosustrophine

Nosustrophine, a biological extract and reported epinutraceutical bioproduct, was produced from domestic pigs (*Sus scrofa domesticus*) brain using non-denaturing biotechnological methods (Patent ID: P202230047/ES2547.5). For more details on nutrition analysis as well as Catecholamines, serotonin, L-dopa, and neurotrophic factors analysis, see previous publication [15] and Supplementary Materials.

### 4.3. Experimental Design

This novel triple *Bin1*/*Cops5*/*App* transgenic mice were generated by a crossbreeding strategy to obtain mice at different stages of development. Thus, triple transgenic mice with 3 and 9 months of age were generated and euthanized, together with wild-type mice used as control groups. The EB/3xTg-AD mice, once generated and genotyped by PCR, were randomly divided into these four experimental groups by age (Figure 1), as follows: Group A (3–4 months of age) comprised 12 mice (9 transgenic and 3 wild-type mice); group B (8–9 months of age) contained 12 mice (9 transgenic and 3 wild-type mice); and group D (8–9 months of age) comprised 9 wild-type mice. Mice were housed in a room with controlled temperature (20–21 °C), humidity (40–50%), and lighting (12 h light/dark cycle) and were supplied with water ad libitum. The diets/treatment regime was performed for four weeks. 

Treatment origin: Nosustrophine is a nootropic supplement made by multi-peptide complex extracted from young porcine brain (*Sus scrofa* ssp.) by non-denaturing biotechnological processes which preserve the natural properties of its active neurotrophic ingredients.

Treatment preparation: Nosustrophine extract (young porcine brain extract) was integrated into the diet as pellet biscuits elaborated in our laboratory by adding 50% of powder treatment ingredients (Nosustrophine) using diet wheat as the main flour and adding 10% (*w*/*w*) Milli-Q purified water for pelleting and then drying the pellets at 34 °C overnight. The principle nutrient composition of the treatment diet is shown in Table 1.

### 4.4. Immunohistochemistry and Motor Function Evaluation

Immunohistochemical hallmarks and motor function performance were analyzed as previously published [16,17]. For more details, see Supplementary Materials.

### 4.5. Imaging

Fluorescence signals were captured with a Leica DM6-B upright microscope (Leica Microsystems, Buffalo Grove, IL, USA) and Leica Application Suite X (LAS X) software (version 3.7.6.25667). For more details, see Supplementary Materials.

### 4.6. Statistical Analysis

Statistical analysis was performed with SPSS v. 11.0 (SPSS, Inc., Chicago, IL, USA). Differences between treated groups were compared using the Kruskal–Wallis test followed by a Mann–Whitney U test for non-parametric data, while normally distributed data were tested using a one-way analysis of variance (ANOVA). Scheffé correction was used to compare combinations of treatment groups. Data are expressed as standard error of the mean (±SEM). Statistically significant differences are indicated as follows (* *p* < 0.05, ** *p* < 0.01, or *** *p* < 0.001).

## 5. Conclusions

Nosustrophine may be able to successfully lessen the cognitive impairment and pathological and behavioral abnormalities brought on by the hallmarks of AD in 3xTg-AD mice. We demonstrate here that Nosustrophine is neuroprotective, corresponding with the improvement of the pathologic hallmarks of AD, even though further research is still needed to fully understand the molecular pharmacodynamics of the extract. The neurotrophic cocktail in Nosustrophine also appears to be essential to the molecular mechanisms of neuroprotection against brain degeneration, notably in the hippocampus and neocortex, in addition to its involvement in brain development, physiology, and disease. The benefits of neurotrophic factors produced from pig extract on degenerative disorders have been the subject of several preclinical investigations; however, these reports do not guarantee clinical efficacy against each distinct characteristic.

## Figures and Tables

**Table 1:** Nutritional composition of both normal and Nosustrophine-containing diets. The nutritional composition of the Nosustrophine-containing diet used in the experiment showed more protein and fat content when compared with normal diet. Values were rated per 100 g.

	**Normal Diet**		**Nosustrophine**	
	**(g)**	**(% kcal)**	**(g)**	**(% kcal)**
Protein	18.6	20	48	33
Carbohydrates	68.2	70	0	0
Fat	4.4	10	44	67
*Saturated*	(2)	-	(18.9)	-
*Monosaturated*	(1.3)	-	(14.5)	-
*Polyunsaturated*	(1.1)	-	(10.6)	-
Total	91.2	100	92	100

**Table 2:** Antibodies used for immuno-histochemistry/fluorescence.

Antibody	Antigen	Type	Source	Dilution	Ref.
β-Amyloid (IHC)	Aβ1-42 (mouse)	Mouse monoclonal	Millipore	1:600	05-831-I
β-Amyloid (IF)	Aβ1-42 (rabbit)	Rabbit polyclonal	Invitrogen	1:500	51-2700
GFAP (IHC)	Glial fibrillary acidic protein (mouse)	Mouse monoclonal	Sigma	1:100	G-3893
CD11b (IHC)	CD11/B-cells (rat)	Rat polyclonal	Millipore	1:500	MABF514
TH (IF)	Tyrosine Hydroxylase (mouse)	Mouse monoclonal	Millipore	1:500	IHCR1005-6
NeuN (IF)	Neuronal Nuclei (mouse)	Mouse monoclonal	Millipore	1:500	MAB377
Cox-2 (IF)	Cyclooxygenase 2 (rabbit)	Mouse monoclonal	Vector	1:400	PA5-96081
IL-17 (IF)	Interleukin 17 (rat)	Rat polyclonal	Invitrogen	1:500	12-7177-81

## Data Availability

Data is contained within the article and supplementary material.

## Supplementary Materials

The following supporting information can be downloaded at: https://www.mdpi.com/article/10.3390/ph16091306/s1; Table S1: Composition of Nosustrophine. References [41,42,43,44] have been cited in supplementary materials.
