# Supplementary material for: Neuroprotective Effect of Nosustrophine in a 3xTg Mouse Model of Alzheimer’s Disease
Source: Pharmaceuticals (Basel). 2023 Sep 15;16(9):1306. doi: 10.3390/ph16091306 (PMC10535028; doi:10.3390/ph16091306)
Supplement: Supplementary file 1 [file pharmaceuticals-16-01306-s001.zip › pharmaceuticals-2565846-supplementary.pdf]

SUPPLEMENTARY DATA

Material and Methods

Mouse models

EB/3×Tg-AD (*App*/*Bin1*/*Cops5*) mice were generated and used in the present research study. This triple transgenic mouse AD model, which overexpresses the Swedish mutation of *APP* (human amyloid precursor protein) together with *BIN1* (bridging integrator 1, AMPH2) and *COPS5* (COP9 constitutive photomorphogenic homolog subunit 5, Jab1) closely mimics the human brain pathology. DNA constructs and transgenic generation proceedings have been described previously [15, 16] and were sequence-verified prior to breeding the transgenic colony. Transgenic mice were identified by PCR analysis of tail DNA as described previously [15, 16]. In detail, the *Cops5* cDNA was PCR amplified using the following primers: forward, 5′-cggaattcatggcggcgtccgggagcggt-3′; reverse, 5′-gcgtcgacttaagagatgttaatttgatt-3′. The *Bin1* cDNA was PCR amplified using the following primers: forward, 5′-cggaattcatggcggcgtccgggagcggt-3′; reverse, 5′-gcgtcgacttaagagatgttaatttgatt-3′, while the *App* cDNA was PCR amplified using the following primers: forward, 5′-cggaattcatggcggcgtccgggagcggt-3′; reverse, 5′-gcgtcgacttaagagatgttaatttgatt-3′. Mice were generated by crossing heterozygous line 1903 of *Cops5*-Tg mice with the heterozygous *Bin1*-Tg and *APP*swe-transgenic mice. The microinjection of cloned and linearized cDNA in to the blastocyst was carried out at the Sylvester Comprehensive Cancer Center Transgenic Core Facility (University of Miami) using standard techniques by strictly following animal use protocols as approved by the Animal Care and Use Committee at the Torrey Pines Institute for Molecular Studies in accordance with National Institutes of Health guidelines. All experimental mice procedures were conformed to the guidelines established by the European Communities Council Directive (86/609/EEC), the EU Directive 2010/63/EU, and the Spanish Royal Decree 1201/2005 for animal experimentation and were approved by the Ethical Committee of the EuroEspes Biotechnology Research Centre (Permit number: EE/2017-127).

Biochemical characterization of Nosustrophine

Nosustrophine is a biological extract and reported as an epinutraceutical bioproduct that was obtained through non-denaturing biotechnological processes from the domestic pig (*Sus scrofa domesticus*) brain (Patent ID: P202230047/ES2547.5).

Nutritional analysis: The powdered extract was analyzed for its nutritional composition. This analysis was outsourced to a laboratory specialized in feeding and characterization of raw materials (Alkemi (AGQ Lab Group, Madrid, Spain). A basic evaluation was included: total fat, saturated, monounsaturated and polyunsaturated fats, trans-fatty acids, starch, ashes, sodium chloride, cholesterol, dietary fiber, carbohydrates, humidity, proteins and energy value. The Nosustrophine composition is already published elsewhere [35]. Subsequently, a detailed analysis of each of the nutrient groups was performed. Quantification of 38 fatty acids was determined by gas chromatography with flame ionization detector (FID). High performance liquid chromatography (HPLC) with ultraviolet-visible (UV-Vis) detector was used to measure 11 sugars (fructose, galactose, glycerol, glucose, lactitol, lactose, maltitol, maltose, mannitol, sucrose and sorbitol), 11 amino acids, B-complex vitamins, vitamin C, D and E. Nutritional minerals were analyzed by flame atomic absorption except for calcium which was used UV-V spectrophotometry. For heavy metals, graphite furnace atomic absorption spectroscopy was used for cadmium determination and inductively coupled plasma mass spectrometry (ICP-MS) for total selenium.

Catecholamines, serotonin and L-dopa analysis: Fractionated catecholamines and serotonin were measured by Ultra High-Performance Liquid chromatography (UHPLC) method with electrochemical detection (ECD) in the ALEXYS Neurotransmitter Analyzer (Antec Scientific, USA) [41,42]. Prior to HPLC injection, the powder was dissolved in PBS 1x and homogenized in the vortex. It was centrifuged at 4000 rpm and the supernatant was collected for analysis. A specific sample clean-up was performed using a solid phase extraction for catecholamines analysis and a precipitation step in case of serotonin to remove the sample matrix and to spike with an internal standard. Histamine concentration was measured by HPLC, that consists of four independent isocratic pumps, a stainless steel column packed with a cation exchanger (TSK gel SP-2SW, 5 μm; TosoHaas Corporation) and a fluorometric detection system (FLD). Samples of 20 μl were injected directly into the HPLC column. The HPLC method is based on the extraction of histamine with perchloric acid, followed by direct HPLC analysis with on-line derivatization with ophthaldialdehyde and fluorescence detection, setting the excitation wavelength at 360 nm and the emission at 450 nm, as previously described [43]. Histamine, catecholamines and serotonin were expressed as pg/mg of whole extract. L-dopa levels was measured by HPLC-UV using a Varian 920-LC analyzer (Agilent Technologies, USA) after extraction with perchloric acid 5% and subsequent filtering of the supernatant through a membrane of 0.45 μm pore size and 13 mm diameter (Adventec Dismic-13NP Nylon Cat. No. 13NP045AN) using 2.5 mL syringes (BD plastipack) according to the method described by Burbano et al. (1995) [44]. The results were expressed as mg/g of whole extract. These analyzed compounds are thermosensitive therefore the sample was kept in an ice bath throughout the extraction process. L-DOPA analysis was performed by URIKER laboratory (Bizkaia, Spain). For more details see reported data [15].

FATTY ACIDS	%	METHOD
Oleic acid	28.1	GC-FID
Stearic acid	28	GC-FID
Docosahexaenoic acid	14	GC-FID
Arachidonic acid	13.6	GC-FID
Palmitic acid	11	GC-FID

AMINO ACIDS	%	METHOD
Glutamic acid	6	HPLC UV-Vis
Aspartic	5.1	HPLC UV-Vis
Leucine	4.4	HPLC UV-Vis
Arginine	3.7	HPLC UV-Vis
Lysine	3.4	HPLC UV-Vis
Serine	3.4	HPLC UV-Vis
Alanine	2.9	HPLC UV-Vis
Glycine	2.7	HPLC UV-Vis
Phenylalanine	2.6	HPLC UV-Vis
Proline	2.3	HPLC UV-Vis
Valine	2.2	HPLC UV-Vis
Threonine	2.1	HPLC UV-Vis
Thyroxine	2.1	HPLC UV-Vis
Isoleucine	1.7	HPLC UV-Vis
Histidine	1.4	HPLC UV-Vis
Cysteine	0.73	HPLC UV-Vis
Methionine	0.6	HPLC UV-Vis
Tryptophan	0.55	HPLC UV-Vis

VITAMINS	mg/kg	METHOD
B2	82	HPLC UV-Vis
B3	17	HPLC UV-Vis
D3	8.1	HPLC UV-Vis
E	184	HPLC UV-Vis

CARBOHYDRATES	g/100 g	METHOD
Fructose	< 0.10	HPLC UV-Vis
Galactose	< 0.10	HPLC UV-Vis
Glycerol	< 0.10	HPLC UV-Vis
Glucose	< 0.10	HPLC UV-Vis
Lactitol	< 0.10	HPLC UV-Vis
Lactose	< 0.10	HPLC UV-Vis
Maltitol	1	HPLC UV-Vis
Maltose	< 0.10	HPLC UV-Vis
Manitol	< 0.10	HPLC UV-Vis
Saccharose	< 0.10	HPLC UV-Vis
Sorbitol	4.15	HPLC UV-Vis
Total polyalcohols	5.2	

NUTRITIONAL MINERALS	mg/kg	METHOD
Calcium	503	HPLC UV-Vis
Magnesium	93	FAAS
Zinc	60	FAAS
Iron	52	FAAS
Copper	17	FAAS
Potassium	12.799	FAAS
Manganese	3.9	FAAS
Selenium	1.06	ICP-MS

OTHER COMPOUNDS		METHOD
L-DOPA	22.5 mg/g	HPLC-UV
Dopamine	2760.4 pg/mg	UHPLC-ECD
Noradrenaline	655.4 pg/mg	UHPLC-ECD
Serotonin	479 pg/mg	UHPLC-ECD
Histamine	158.5 pg/mg	HPLC-FLD
BDNF	35 pg/mg	ELISA
Adrenaline	20 pg/mg	UHPLC-ECD
Somatostatin	7.75 pg/mg	RIA
CRF	0.658 pg/mg	ELISA

Table S1. Composition of Nosustrophine (previously publicated [15]. BDNF, brain-derived neurotrophic factor; CRF, corticotropin-releasing factor; ELISA, enzyme-linked immunosorbent assay; FAAS, flame atomic absorption spectrometry; GC-FID, gas chromatography with flame-ionization detection; HPLC UV-Vis; high performance liquid chromatography with ultraviolet-visible; ICP-MS, inductively coupled plasma mass spectrometry; L-DOPA, 3,4-dihydroxyl-L-phenylalanine; RIA, radioimmunoassay; UHPLC-ECD; ultra-high-performance liquid chromatography with electrochemical detection.

Neurotrophic factors: The levels of trophic factors BDNF (Brain-Derived Neurotrophic Factor) and CRF (Corticotropin Releasing Factor) were analyzed by commercial multi-species enzyme immunoassay (ELISA) and those of Somatostatin (Growth Hormone Inhibiting Hormone) by radioimmunoassay. For

more details see reported data [15].

Neurobiochemical effect of Nosustrophine in EB/3×Tg-AD mice serum
Blood collection was performed by cardiac puncture after deep anesthesia of the mouse immediately before sacrifice. Commercial Greiner Bio-One brand tubes without anticoagulant were used. These tubes were centrifuged at 4000 rpm for 10 min to separate the serum from the cell layer, after allowing them to clot for 20 min in an upright position after extraction. The serum was stored at -80 °C until the analysis of the following parameters: albumin, calcium, phosphorus, magnesium, iron, creatinine, GOT/GPT, total anti-oxidant capacity (TAS), glutathione reductase by UV-visible spectrophotometry, and reactive C protein by turbidimetric technique, all of them in the Cobas Mira Plus analyzer (Roche Diagnostics, Basel, Switzerland). Vitamins B6 and B9 levels were measured with commercial ELISA kits from Elabscience (USA). For the determination of malondialdehyde (MDA), the TBA method (colorimetric technique) was used (Elabscience, USA). The reactions were read by absorbance in the EPOCH reader (Biotek Instruments, USA).

Immunohistochemistry
Parallel transverse sections (12–14 µm) from the left half of the brain were obtained with a cryostat and pretreated with H_2O_2 in phosphate-buffered saline (PBS) at room temperature for 15 min to eliminate endogenous peroxidase. Then, rinsed twice in 0.05M Tris-buffered saline (TBS) containing 0.1% Tween-20 at pH 7.4 (TBS-T) for 10 min each, pre-treated with blocking avidin/biotin kit (Vector) and then incubated overnight with the primary antibodies as described in table 2. The sections were successively rinsed in TBS-T, incubated in goat IgG anti-rat (Millipore) or goat IgG, anti-mouse (Sigma), depending on the primary antibody, for 1 hour, rinsed in TBS-T, and then incubated for 30 min using the ABC kit (Vectastain; Vector). The labeling was revealed by incubating sections with 3,3-diaminobenzidine (DAB, Sigma) as the chromogen and hydrogen peroxide as the oxidant. In several adjacent sections, negative controls performed by omitting the primary, secondary, or tertiary antibodies showed no immunostaining. The neuropathological markers observed (Aβ, GFAP, CD11) were digitally quantified together with behavior test results (rotarod).

Immunofluorescence hallmarks were analyzed by performing the same preparation steps as mentioned before. Then the sections were subsequently incubated with primary antibodies against neuronal nuclear protein (NeuN), β-amyloid plaques (Aβ), apoptotic marker (cyclooxygenase 2, Cox2), proinflammatory cytokine (interleukin 17, IL17) and a marker of dopaminergic neurons (tyrosine hydroxylase, TH) in blocking solution over-night at 4 °C. Negative primary controls included the blocking solution only. The tissues were washed in PBS and then incubated for 2 h at RT with a secondary antibodies (Alexa-488/594/350-conjugated; Thermo Fisher, Scientific,Waltham, MA, USA) diluted in blocking solution. The sections were then rinsed three times for 15 min in PBS, and coverslipped with Vectashield medium (Vector Labs, Newark, CA, USA). Fluorescence signals were captured with a Leica DM6-B upright microscope (Leica Microsystems, Buffalo Grove, IL, USA) and Leica Application Suite X (LAS X) software. In each tissue section, the areas occupied by immunofluorescence staining in the CA1 subfield and dentate gyrus (DG) of the hippocampus and in the VTA, respectively, were computed with LAS X software from three acquired z-stack datasets per antigen. Wild-type mice used as controls (PBS) was included in figures, in order to stablish a functional immunofluorescence pattern distribution of the immunomarkers performed.

Evaluation of motor function
The motor strength, ability, balance and coordination skills of all the experimental mice were tested on rotarod motor coordination tests at the end of each treatment week. Data are shown in Figure 4 and are presented that allows for comparison with previously published work [13, 16]. Briefly, mice at 3- and 9-months-of-age were habituated to the rotarod apparatus (Columbus Instruments, Columbus, OH) operated from 10-40 rpm over a period of 60 seconds for four consecutive days with five repetitions of the task, separated by a rest period of 15 min (each day), sufficient to reach a baseline level of performance. The fifth day constituted the test day and the mice were tested on the same experimental paradigm for four trials/weeks. The average latency to fall was plotted for each group and for each of

those four test trials. This procedure was designed to assess motor coordination within 30 days. The latency to fall off the rotarod was recorded. The time each mouse remained on the rotarod was registered automatically and stopped when the animal fell or inverted (by climbing) from the top of the rotating barrel. Observers were blinded to animal conditions.
